# Integrating cell cycle score for precise risk stratification in ovarian cancer

**DOI:** 10.3389/fgene.2022.958092

**Published:** 2022-08-17

**Authors:** Lingying Chen, Haiyan Gu, Lei Zhou, Jingna Wu, Changdong Sun, Yonggui Han

**Affiliations:** ^1^ Department of Obstetrics and Gynecology, Beilun District People’s Hospital, Ningbo, China; ^2^ Department of Obstetrics and Gynecology, Beilun No 3 People’s Hospital, Ningbo, China

**Keywords:** ovarian cancer, cell cycle, mesenchymal subtype, prognosis, PD-1/PD-L1

## Abstract

**Background:** Ovarian cancer (OC) is a highly heterogeneous disease, of which the mesenchymal subtype has the worst prognosis, is the most aggressive, and has the highest drug resistance. The cell cycle pathway plays a vital role in ovarian cancer development and progression. We aimed to screen the key cell cycle genes that regulated the mesenchymal subtype and construct a robust signature for ovarian cancer risk stratification.

**Methods:** Network inference was conducted by integrating the differentially expressed cell cycle signature genes and target genes between the mesenchymal and non-mesenchymal subtypes of ovarian cancer and identifying the dominant cell cycle signature genes.

**Results:** Network analysis revealed that two cell cycle signature genes (*POLA2* and *KIF20B*) predominantly regulated the mesenchymal modalities of OC and used to construct a prognostic model, termed the Cell Cycle Prognostic Signature of Ovarian Cancer (CCPOC). The CCPOC-high patients showed an unfavorable prognosis in the GSE26712 cohort, consistent with the results in the seven public validation cohorts and one independent internal cohort (BL-OC cohort, qRT-PCR, *n* = 51). Functional analysis, drug-sensitive analysis, and survival analysis showed that CCPOC-low patients were related to strengthened tumor immunogenicity and sensitive to the anti-PD-1/PD-L1 response rate in pan-cancer (*r* = −0.47, OC excluded), which indicated that CCPOC-low patients may be more sensitive to anti-PD-1/PD-L1.

**Conclusion:** We constructed and validated a subtype-specific, cell cycle-based prognostic signature for ovarian cancer, which has great potential for predicting the response of anti-PD-1/PD-L1.

## Introduction

Ovarian cancer (OC) is the leading cause of cancer death in women (Siegel et al., 2019). Due to lack of effective early screening methods and lack of obvious symptoms, most of the patients were diagnosed at an advanced stage, resulting in an overall 5-year survival rate of less than 50% (Lheureux et al., 2019). Clinical risk assessment factors include tumor stage, tumor grade, histopathological classification, de-bulking status, etc. Despite the good initial treatment effect, most ovarian cancer patients still suffer from tumor recurrence and eventually develop drug resistance to chemotherapy (Coleman et al., 2013). Currently, serum CA-125 level is a clinical biomarker for risk assessment of ovarian cancer. Due to its low specificity, the overall assessment effect is not as expected (Bottoni and Scatena, 2015). The high degree of heterogeneity and aggressiveness of OC often leads to treatment failure (Cancer Genome Atlas Research Network, 2011; Konecny et al., 2014). Therefore, there is a need to integrate tumor heterogeneity to identify novel prognostic predictors for OC.

Gene expression-based biomarkers for cancer risk assessment have been extensively explored (Mo et al., 2020). Several studies have established OC prognostic biomarkers based on gene expression (Pan and Ma, 2020; Yang et al., 2021). However, due to the heterogeneity of OC, most of the biomarkers have low prognostic efficacy and cannot be directly used in clinical practice. Recently, four ovarian cancer molecular subtypes with distinct molecular and clinical characteristics were found (Cancer Genome Atlas Research Network, 2011), among which, the mesenchymal subtype had the poorest prognosis. Afterward, the mesenchymal subtype is consistent in several other subtyping systems (Konecny et al., 2014; Chen et al., 2018). Importantly, the mesenchymal subtype of OC shows poor clinical outcomes, indicating the need to integrate the intrinsic modalities of this malignant subtype for risk management in OC.

Cancer manifests itself as an infinite proliferation of cells, the main reason for which is related to improper cell cycle regulation (Williams and Stoeber, 2012). The cell cycle is precisely regulated by cyclin-dependent kinases (CDKs) (Bertoli et al., 2013). However, relevant cell cycle-based biomarkers are rare and still lacking in ovarian cancer. Considering the highly heterogeneous nature of OC, by integrating mesenchymal modalities and the cell cycle signature underlying the mesenchymal subtype, a network-based approach was adopted to identify the dominant cell cycle signature, which regulates the most aggressive OC subtype. Subsequently, we established a prognostic model, termed Cell Cycle Prognostic Signature of Ovarian Cancer (CCPOC), and exploration of the prognosis capacity of CCPOC in OC. Our signature incorporates cell cycle system and tumor heterogeneity and would be used to screen OC patients who may benefit from a more precise treatment.

## Materials and methods

### Public dataset preparation and preprocessing

We obtained 1,798 OC samples from eight publicly available datasets. The training dataset was the GSE26712 (Bonome et al., 2008) cohort (*n* = 182). Validation cohorts were the TCGA(Cancer Genome Atlas Research Network, 2011) (*n* = 578), GSE9891 (Tothill et al., 2008) (*n* = 285), ICGC-AU (Patch et al., 2015) (*n* = 111), GSE138866 (Hu et al., 2020) (*n* = 130), GSE32062 (Yoshihara et al., 2012) (*n* = 260), GSE14764 (Denkert et al., 2009) (*n* = 80), and GSE51088 (Karlan et al., 2014) (*n* = 172) datasets. Together with the corresponding clinical information, the normalized expression datasets sourced from the GEO database were downloaded *via* the GEOquery package (version 2.58.0). The transcription data (Affymetrix U133A) and relevant clinical information on TCGA were retrieved from the Firebrowse (http://firebrowse.org/) database. The standardized expression profile and clinical information of ICGC-AU were downloaded from the International Cancer Genome Consortium (ICGC, https://icgc.org) OV-AU (Ovarian cancer-Australia) database. For the microarray data, the gene expression data probe IDs were transformed into gene symbols; if multiple probe IDs were mapped to the same gene symbol, the one with the highest average value was selected. The molecular subtyping information was retrieved from Verhaak’s study (Verhaak et al., 2010). The detailed clinical parameters of all cohorts are listed in Table 1.

**TABLE 1:** Overview of the clinical and pathologic characteristics of all the datasets.

	Training cohort	Public validation cohorts							Internal validation
	GSE26712	TCGA	GSE9891	ICGC-AU	GSE138866	GSE32062	GSE14764	GSE51088	BL-OC
Number of patients	182	578	285	111	130	260	80	172	51
Age (years)									
Mean, years (STD)	62 (11.9)	60 (11.6)	60 (10.6)	59 (8.7)	62 (11.9)			58 (12.6)	58 (11.2)
Histopathology									
Serous	182 (100%)	568 (98%)	264 (93%)	111 (100%)	130 (100%)	260 (100%)	68 (85%)	122 (71%)	58 (100%)
Others		10 (2%)	21 (7%)				12 (15%)	50 (29%)	
Stage									
I		16 (3%)	24 (8%)				8 (10%)	22 (13%)	
II		27 (5%)	18 (6%)		2 (2%)		1 (1%)	9 (5%)	
III	144 (79%)	436 (75)	218 (76%)	96 (86%)	106 (82%)	204 (78%)	69 (86%)	103 (60%)	
IV	36 (20%)	84 (15%)	22 (8%)	15 (14%)	22 (17%)	56 (22%)	2 (3%)	17 (10%)	
Unknown	2 (1%)	15 (3%)	3 (1%)						
Grade									
Well		6 (1%)	19 (7%)				3 (4%)	16 (9%)	
Moderately		69 (12%)	97 (34%)	15 (14%)		131 (50%)	23 (29%)	14 (8%)	
Poorly		479 (83%)	163 (57%)	66 (59%)	130 (100%)	129 (50%)	54 (68%)	119 (69%)	
Unknown		23 (4%)	6 (2%)	30 (27%)				23 (13%)	
Debulking									
Optimal	88 (48%)	367 (63%)	160 (56%)		107 (82%)	103 (40%)	39 (49%)		
Suboptimal	94 (52%)	140 (24%)	88 (31%)		15 (12%)	157 (60%)	23 (29%)		
Unknown		71 (12%)	37 (13%)		8 (6%)		18 (22%)		
Vital status									
Alive	55 (30%)	270 (47%)	169 (59%)	23 (21%)	31 (24%)	139 (53%)	59 (74%)	40 (23%)	16 (31%)
Dead	127 (70%)	290 (50%)	113 (40%)	88 (79%)	99 (76%)	121 (47%)	21 (26%)	112 (65%)	35 (69%)
NA		18 (3%)	3 (1%)					20 (12%)	
Median OS, months (±SE)	38.2 (2.6)	29.4 (1.1)	28.5 (1.4)	32.4 (3.0)	33.7 (4.6)	41.5 (1.5)	35.0 (1.7)	49.7 (4.0)	37.5 (4.6)

### Clinical samples

For the independent internal validation cohort (BL-OC cohort), we retrospectively collected 51 formalin-fixed paraffin-embedded (FFPE) blocks from patients who underwent surgery in Beilun People’s Hospital (from 1st January, 2015 to 1st January, 2021), Ningbo, China. Criteria for patient sample selection: longer follow-up (> 5 years) and had evaluation of adjuvant chemotherapy efficacy and no history of cancer other than ovarian cancer. This study was approved by the Ethics Committee of the Beilun People’s Hospital.

### Network analysis screening key regulated cell cycle genes for the mesenchymal subtype

We obtained 313 cell cycle-related genes (CRGs) through the concatenation of the cycle-related genes from the MSigDB database (Version 7.2; KEGG cell cycle pathway, HALLMARK G2M pathway) and Cuzick’s study (Cuzick et al., 2011). CRGs with expression in all datasets were retained for subsequent analysis. We integrated differentially expressed target genes and cell cycle genes between the mesenchymal subtype and other subtypes and performed a network analysis by using the RTN package (version 2.10.0) to infer and investigate the relationship between cell cycle genes and potential target genes. Specifically, the network analysis consists of three parts: first, the mutual information (MI) between a cell cycle signature gene and all potential target genes is calculated, and insignificant associations are removed by permutation analysis; second, unstable interactions are removed by bootstrapping; and finally, the ARACNe algorithm is applied to reduce redundant indirect regulations. Together, the GSE26712 dataset was used as the training cohort. Univariate Cox regression analysis screened 34 cell cycle genes (*p* <0.1) for a subsequent analysis. Subsequently, 12 cell cycle genes (|log2 FC| > 0.25, BH-adjusted *p* < 0.05) and 1,704 target genes (log2 FC > 0.25, BH-adjusted *p* < 0.05) were determined differentially expressed in the mesenchymal subtype compared with non-mesenchymal subtypes. Then, a master regulator analysis (Fletcher et al., 2013) (MRA) was performed to examine the overrepresentation of the mesenchymal signature in the regulation of each cell cycle gene by a hypergeometric test. After the hypergeometric test results for all cell cycle signature genes, adjusted *p*-values were calculated using the Benjamini–Hochberg procedure. Two cell cycle signature genes of top significance (Benjamini–Hochberg-adjusted *p*-value < 0.05) were selected as master regulators. For detailed calculation steps and calculation code, please refer to the Vignettes of the RTN package (bioconductor.org/packages/release/bioc/vignettes/RTN/inst/doc/RTN.html).

### Development and evaluation of the risk model for ovarian cancer in public cohorts

Network analysis revealed that two cell cycle genes (*POLA2* and *KIF20B*) were the key regulators of the mesenchymal subtype, which is the most aggressive subtype of ovarian cancer. Subsequently, the multivariable Cox regression model was used to construct a prognostic signature, termed Cell Cycle Prognostic Signature of Ovarian Cancer (CCPOC), in the GSE26172 cohort with these two signature genes. The risk score formula was constructed based on a linear combination of the expression levels weighted with the regression coefficients: CCPOC = (−0.6527 × *POLA2*) + (0.4975 × *KIF20B*). Based on the upper quantile score of each cohort calculated by the risk score formula, patients were divided into CCPOC-high and CCPOC-low subgroups. The prognostic relevance of CCPOC was evaluated in seven public independent validation datasets (TCGA, GSE9891, ICGC-AU, GSE138866, GSE32062, GSE14764, and GSE51088) with the Kaplan–Meier analysis. Univariate and multivariate analyses were performed with other clinical factors to test whether the CCPOC can be considered an independent prognostic predictor.

### Validation of the signature genes in the internal ovarian cancer cohort by quantitative reverse transcription PCR (qRT-PCR)

Fifty-one OC tissues were obtained from Beilun People’s Hospital. This study was approved by the Ethics Committee of Beilun People’s Hospital. Total RNA was extracted by using the High Pure RNA paraffin kit (Roche Applied Science, Indianapolis, IN) from FFPE tissues of the BL-OC cohort. Reverse transcription was performed with High Capacity cDNA (Thermo Scientific). qRT-PCR was performed with the QuantStudio™ 12 K Flex Real-Time PCR System (Thermo Scientific) according to the manufacturer’s recommended operating conditions. β-Actin was tested for data normalization. The primers of each gene are listed as follows: POLA2: F CAC​CAC​ATC​TGA​CAG​CAT​CAC​G, R CCA​CCT​GTT​CAT​GCT​TAG​CAT​CC; KIF20B: F GCT​GAC​TTT​AAG​GAG​ACT​CTG​CT, R GTG​GCA​CAA​ATG​TCT​TTC​GCT​GC; and β-Actin: F CAC​CAT​TGG​CAA​TGA​GCG​GTT​C, R AGG​TCT​TTG​CGG​ATG​TCC​ACG​T. The expression of each gene was calculated using the log2 (2–ΔΔCT) method.

### Functional analysis

Gene Set Enrichment Analysis (GSEA) was carried out to test the dysregulated pathways in different CCPOC risk groups by using the HTSanalyzeR package (Wang et al., 2011) (version 2.3.5) with 1,000 permutations. Hallmark (h.all.v7.2.symbols.gmt) and KEGG (c2.cp.kegg.v7.2.symbols.gmt) gene sets were downloaded from MSigDB (https://www.gsea-msigdb.org/gsea/msigdb/). Only gene sets with >five genes were included in the analyses. To evaluate the immunobiological difference between different CCPOC risk groups, CIBERSORT (Newman et al., 2015), a de-convolution algorithm, was used to characterize 22 types of immune cell abundance for each sample. For the TCGA-OV mutation, data were downloaded from the cBioPortal database (https://www.cbioportal.org/).

### Data sources for chemotherapy, immunotherapeutic, and pan-cancer analysis

GSE146965 (Jiménez-Sánchez et al., 2020) and PMID17290060 (Dressman et al., 2007) containing chemotherapy response information were used for chemotherapy sensitivity analysis. Two immunotherapeutic cohorts: the IMvigor210 cohort (Mariathasan et al., 2018) was an advanced urothelial cancer with the intervention of atezolizumab, an anti-PD-L1 antibody; the GSE78220 cohort (Hugo et al., 2016) was metastatic melanoma treated with pembrolizumab, an anti-PD-1 antibody. For the IMvigor210 cohort, expression data and clinical data were downloaded from https://github.com/SiYangming/IMvigor210CoreBiologies. The TCGA PanCancer Atlas gene expression profiles and clinical information were downloaded by the TCGAbiolinks package (version 2.18.0). The infiltration status of different immune cell populations in the TCGA PanCancer Atlas was downloaded from Tamborero’s study (Tamborero et al., 2018). The marker genes of MHC, immunoinhibitory, and immunostimulatory molecules were reported by Charoentong et al. (2017). DNA damage response (DDR) signature genes were extracted from the study of Theo et al. (Knijnenburg et al., 2018). The expression of the proteins encoded by the signature genes was validated in the Human Protein Profiles (http://www.proteinatlas.org) database. The objective response rate (ORR) was obtained from public research (listed in Supplementary Table S6).

### Statistical analysis

The immune genes and potential target genes between the mesenchymal and non-mesenchymal subtypes underwent differential analysis with the R limma package (version 3.42.2). Kaplan–Meier analysis was performed to test survival differences between different groups with the log-rank test using the R survival package (version 2.41.3). The prognostic value of the selected cell cycle signature was shown using the univariate Cox regression analysis. The independent prognostic effect of CCPOC was tested using univariate and multivariate Cox regression analyses. The survival prediction was assessed by the concordance indices (C-index) and the robust hazard ratio (D-index), which were calculated using the survcomp package. Student’s t-tests and Kruskal–Wallis tests were used to conduct difference comparisons of three or more groups. The correlations between the CCPOC scores and the ORR were evaluated using Pearson’s correlation. *p* < 0.05 was considered significant. All statistical analyses were performed in R (version 3.6.1, *p < 0.05, **p < 0.01, ***p < 0.001).

## Results

### The integrative analysis identifies two cell cycle genes as key regulators in the mesenchymal subtype

**FIGURE 1:**
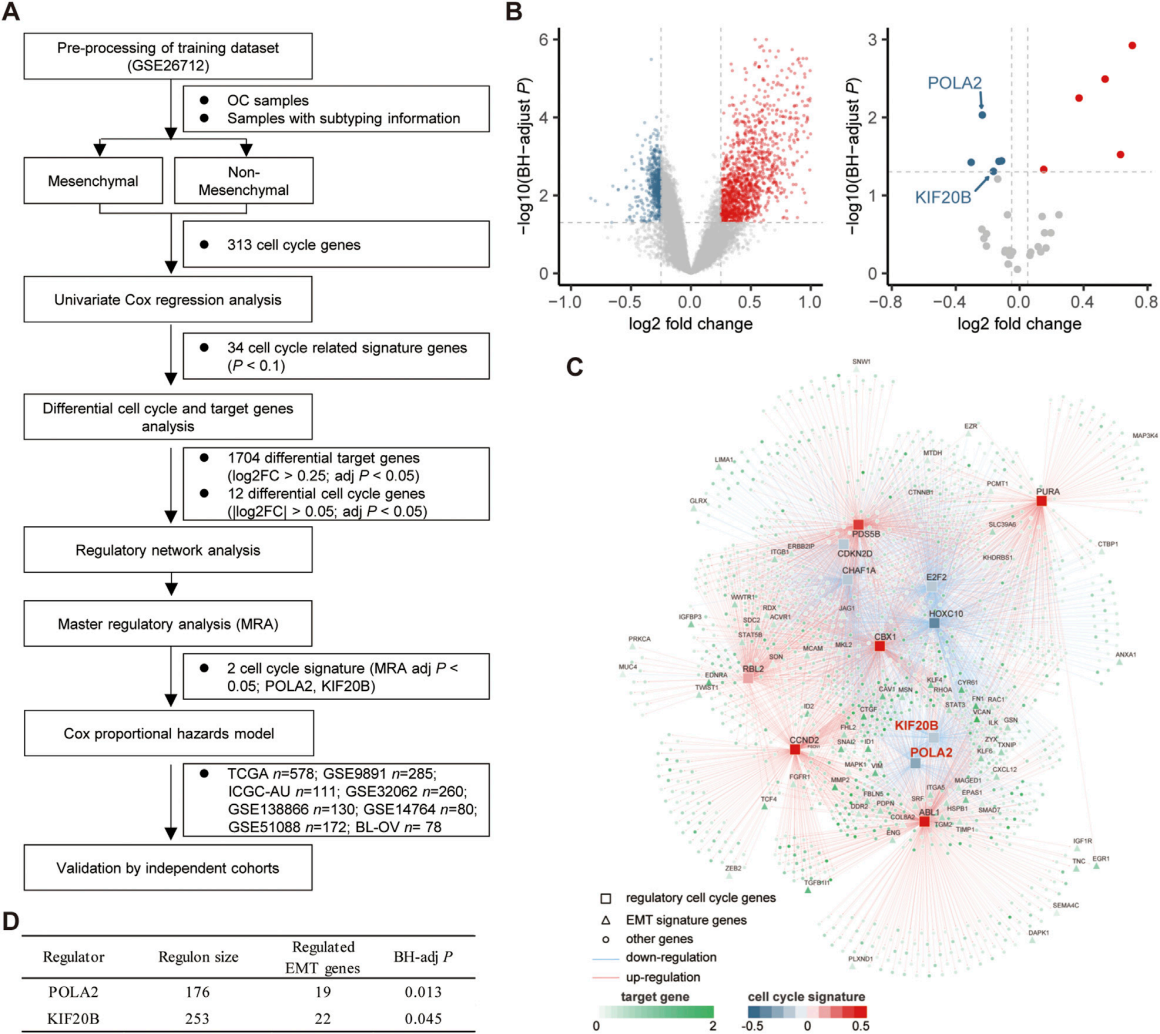
Network inference identified two cycle signature genes (POLA2 and KIF20B) as the key regulators in the mesenchymal subtype of OC. **(A)** Study design of the present work. **(B)** Volcano plot of the differentially expressed target genes and cell cycle signature genes in the mesenchymal subtype. **(C)** Integrated network showing the relationships between the expression data of the cell cycle signature genes and target genes. **(D)** Master regulator analysis results.

The mesenchymal subtype has the worst prognosis and shortest overall survival (Supplementary Figure S1). We intended to integrate the molecular modalities under this subtype to improve the OC risk assessment thereafter. Focusing on the mesenchymal subtype, we applied a network-based approach to investigate the regulatory role of the cell cycle, which is important in the progression of OC. Eight public datasets and one independent internal cohort with a total sample of 1,849 OC cases were included in this study (Table 1). 313 cell cycle-related genes (CRGs) (Supplementary Table S1) were downloaded from the MSigDB database and Cuzick’s study (Cuzick et al., 2011). Based on the GSE26712 cohort, we performed an initial screening of the cell cycle genes by the univariate Cox regression analysis, and a total of 34 cell cycle genes (*p* <0.1) were screened for subsequent analysis. Subsequently, we conducted a differential analysis of the selected cell cycle genes and potential target genes between the mesenchymal and non-mesenchymal subtypes (Figure 1A). Twelve cell cycle genes (|log2 FC| > 0.25, BH-adjusted *p* < 0.05) and 1,704 target genes (log2 FC > 0.25, BH-adjusted *p* < 0.05) were determined to be differentially expressed in the mesenchymal subtype (Figure 1B). Based on the expression profiles of these prioritized cell cycle genes and target genes, we constructed a regulatory network by calculating the mutual information between a cell cycle gene signature and its potential targets (Figure 1C). Based on hypergeometric tests, a master regulator analysis (MRA) was performed to screen core regulators for the mesenchymal subtype (Supplementary Table S2). We identified 19 and 22 EMT genes enriched in the regulons of POLA2 (BH-adjusted *p* = 0.013) and KIF20B (BH-adjusted *p* = 0.045) (Figure 1D), respectively. Compared to the non-mesenchymal subtypes (immunoreactive, proliferative, and differentiated), the two candidate genes were significantly lower expressed in the mesenchymal subtype (Supplementary Figures S2A, B).

**FIGURE 2:**
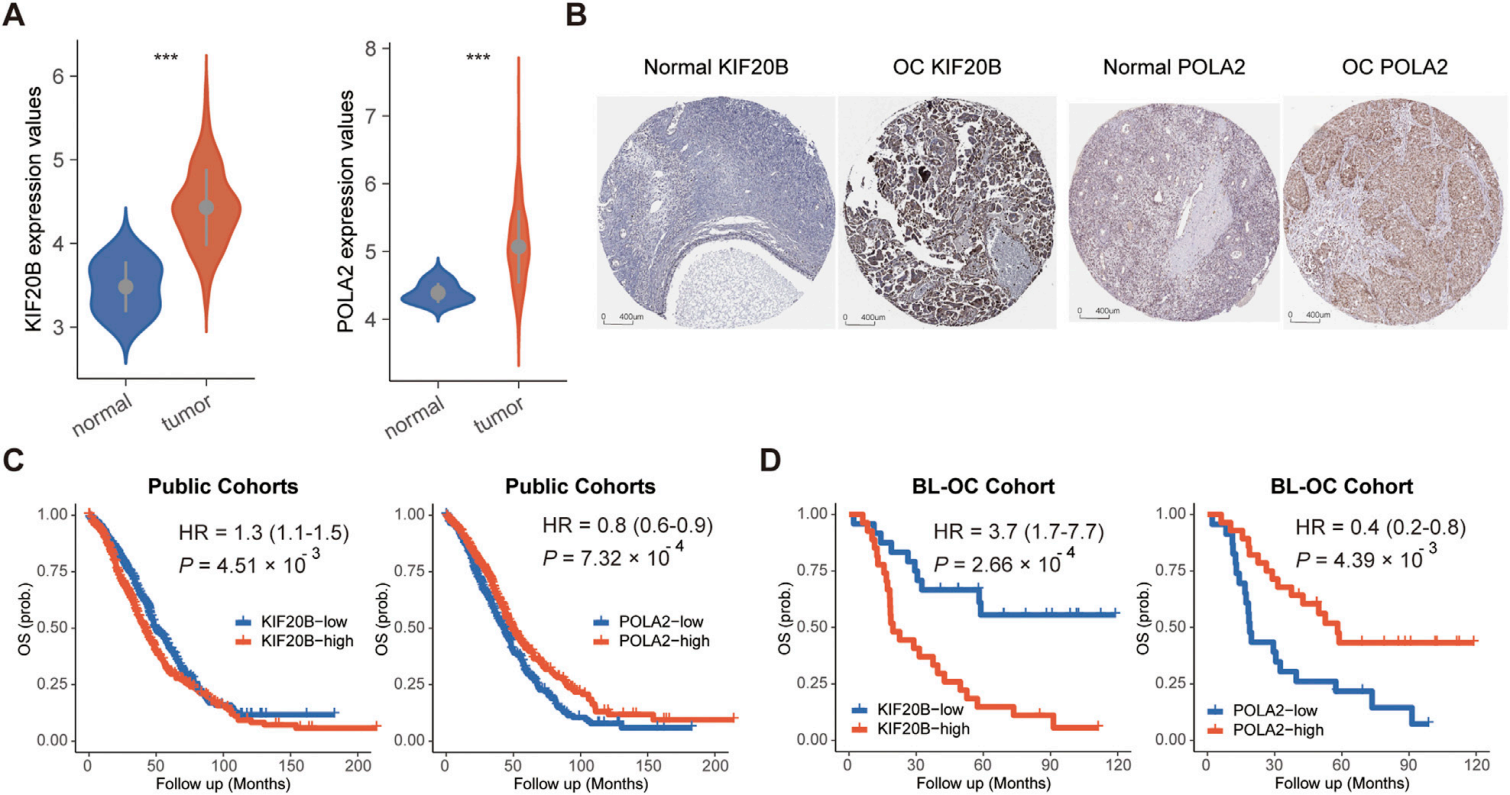
Expression and survival analyses for POLA2 and KIF20B in OC. **(A)** Expression levels of POLA2 and KIF20B in OC and normal tissues. **(B)** Protein levels encoded by POLA2 and KIF20B in normal and OC using clinical samples from the Human Protein Profiles. Survival analysis of POLA2 and KIF20B in public cohorts **(C)** and the BL-OC cohort **(D)**.

Compared to normal tissues, these two candidate cell cycle genes were all significantly highly expressed in OC tissues in the TCGA cohort (Figure 2A). Moreover, we checked the protein levels encoded by these two genes in the Human Protein Profiles database. POLA2 and KIF20B were moderately positive detected in OC clinical specimens when compared to their expression levels in normal samples (Figure 2B). Therefore, in the future, it is possible to evaluate the prognosis of OC patients by detecting the expression of these two genes on clinical specimens by IHC. Furthermore, the survival analysis revealed a prognostic association with overall survival in the public cohorts (Figure 2C) and the BL-OC cohort (Figure 2D). Together, the network-based approach identified two cell cycle genes, with a prognostic value, as key regulators in the mesenchymal subtype.

### Construction and evaluation of the cell cycle prognostic signature in public cohorts and the BL-OC cohort

**FIGURE 3:**
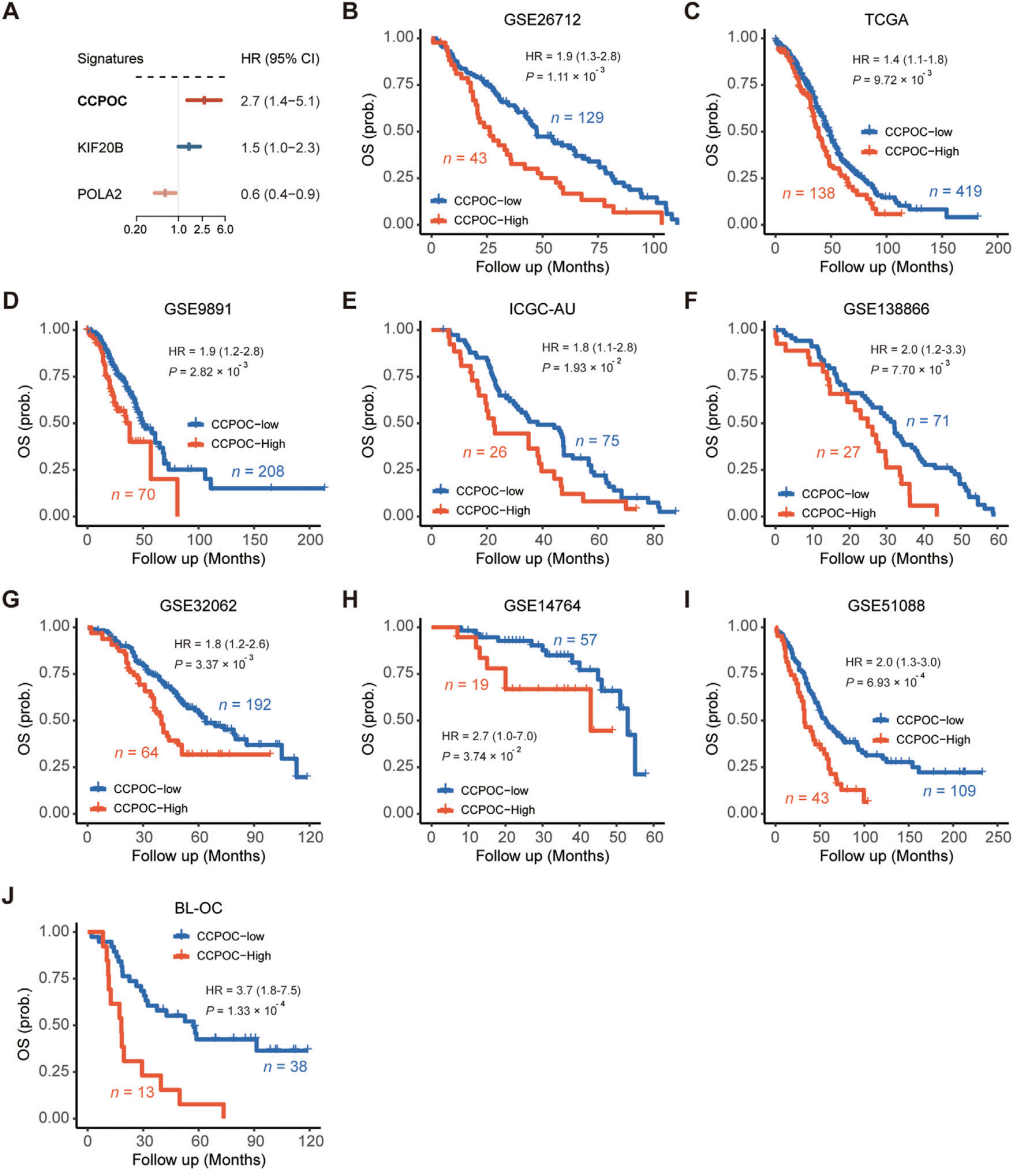
Assessment of the prognostic value of the CCPOC. **(A)** Comparison of prognostic efficiencies between the CCPOC and its individual constituents. **(B)** Kaplan–Meier survival analysis showing that the CCPOC-high group had an unfavorable OS in the training cohort (GSE26712). In the seven public validation cohorts **(C–I)**, the CCPOC-high group stably showed a significantly poor prognosis for OS. **(J)** Evaluation of the prognostic value of CCPOC in the BL-OC cohort.

Based on the GSE26172 cohort, the risk model called “Cell Cycle Prognostic Signature of Ovarian Cancer” (CCPOC) was constructed based on a linear combination of the expression levels weighted with the regression coefficients of these two cell cycle genes derived from the multivariate Cox regression analysis. Risk score = (−0.6527 × *POLA2*) + (0.4975 × *KIF20B*). Subsequently, risk scores were calculated for all patients in the public cohorts and our in-house validation BL-OC cohort (Supplementary Tables S3–S4). The CCPOC score showed prognostic efficiency with an AUC of 0.77 at 2 years and 0.79 at 5 years in the BL-OC cohort (Supplementary Figure S3). Based on the upper quantile score of each cohort calculated by the risk score formula, patients were divided into CCPOC-high and CCPOC-low subgroups. Suboptimal samples were enriched in the CCPOC-high group; meanwhile, KIF20B was highly expressed in the CCPOC-high group, while POLA2 was highly expressed in the CCPOC-low group (Supplementary Figure S4). The CCPOC showed stronger prognostic efficiency than its individual constituents (Figure 3A). In the GSE26172 cohort, patients in the CCPOC-high group had significantly poorer OS than patients (Figure 3B, Supplementary Table S5). Moreover, the CCPOC-high group had significantly reduced OS compared to the CCPOC-low group in the seven public validation cohorts (Figure 3C–I, Supplementary Table S5) and our internal validation BL-OC cohort (Figure 3J, Supplementary Table S5). In addition, the CCPOC remains effective at discriminating survival after adjusting for clinical factors, including sex and de-bulking status (*p* < 0.05, Supplementary Figure S5). To test whether the CCPOC was an independent prognostic predictor, univariate and multivariate Cox regression analyses were conducted in the GSE26172 cohort and meta-validation of public cohorts. After adjusting for the clinicopathological parameters, the CCPOC remained independently prognostic (Table 2). Together, these findings indicated that the CCPOC was an independent prognostic signature.

**TABLE 2:** Univariate and multivariate prognostic analyses of the cell cycle signature and clinicopathological factors in the training cohort and meta-validation of public cohorts.

	GSE26712				Meta-validation of public cohorts			
	Univariate		Multivariate		Univariate		Multivariate	
	HR (95% CI)	*p*	HR (95% CI)	*p*	HR (95% CI)	*p*	HR (95% CI)	*p*
Grade* (3 vs. 1&2)	1.75 (0.44–7.07)	0.43	1.63 (0.40–6.58)	0.49	2.63 (1.52–4.55)	**5.0E-04**	2.09 (0.87–5.05)	0.10
Debulking (optimal vs. suboptimal)	1.32 (1.02–1.71)	**0.03**	1.29 (0.99–1.68)	**0.05**	1.31 (1.10–1.54)	**2.0E-03**	1.27 (1.08–1.51)	**4.0E-03**
CCPOC (high vs. low risk)	1.40 (1.08–1.81)	**0.01**	1.29 (1.00–1.69)	**0.04**	1.61 (1.39–1.88)	**3.4E-10**	1.55 (1.30–1.85)	**1.2E-06**

*One well-differentiated; two moderately differentiated; three poorly differentiated.

Numbers in bold indicate significance of 0.05 or less.

### Comparison with existing prognostic models

**FIGURE 4:**
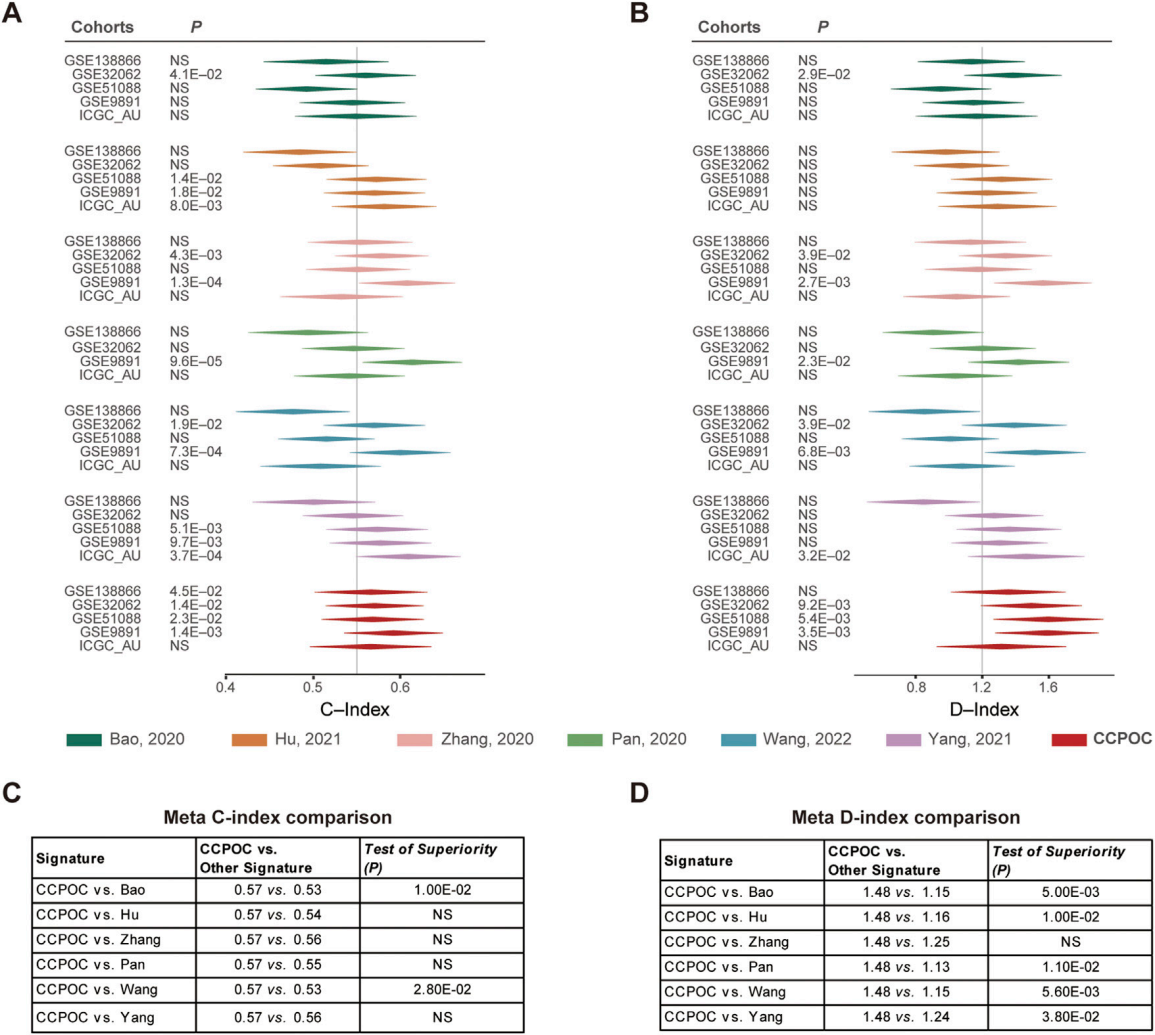
Comparison of published classifiers with CCPOC. Forest plot reporting of the **(A)** concordance index (C-index) and **(B)** D-index (robust hazard ratio) for CCPOC and published classifiers. The tables illustrate the superiority of CCPOC compared with published classifiers for Meta C-index **(C)** and Meta D-index **(D)**.

Next, in order to compare the prognostic value of the CCPOC with published OC prognostic models, referred to as Bao et al. (2020), Hu et al. (2021), Zhang et al. (2021), Pan’s (Pan and Ma, 2020), Qi’s (Ye et al., 2021), Wang et al. (2022), and Yang et al. (2021) prognostic system, based on the OS data of the GSE138866, GSE32062, GSE51088, GSE9891, and ICGC-AU cohorts, the C-index and D-index were calculated. As presented in Figure 4, the C-index was significantly higher in CCPOC than existing Bao and Wang models (Figures 4A, C). Like the C-index, the D-index was significantly higher in CCPOC than in most of the existing prognostic systems (Figures 4B, D).

### Illustrating the immune microenvironment composition, dysregulated pathways, and drug sensitivity in CCPOC-low and CCPOC-high groups

**FIGURE 5:**
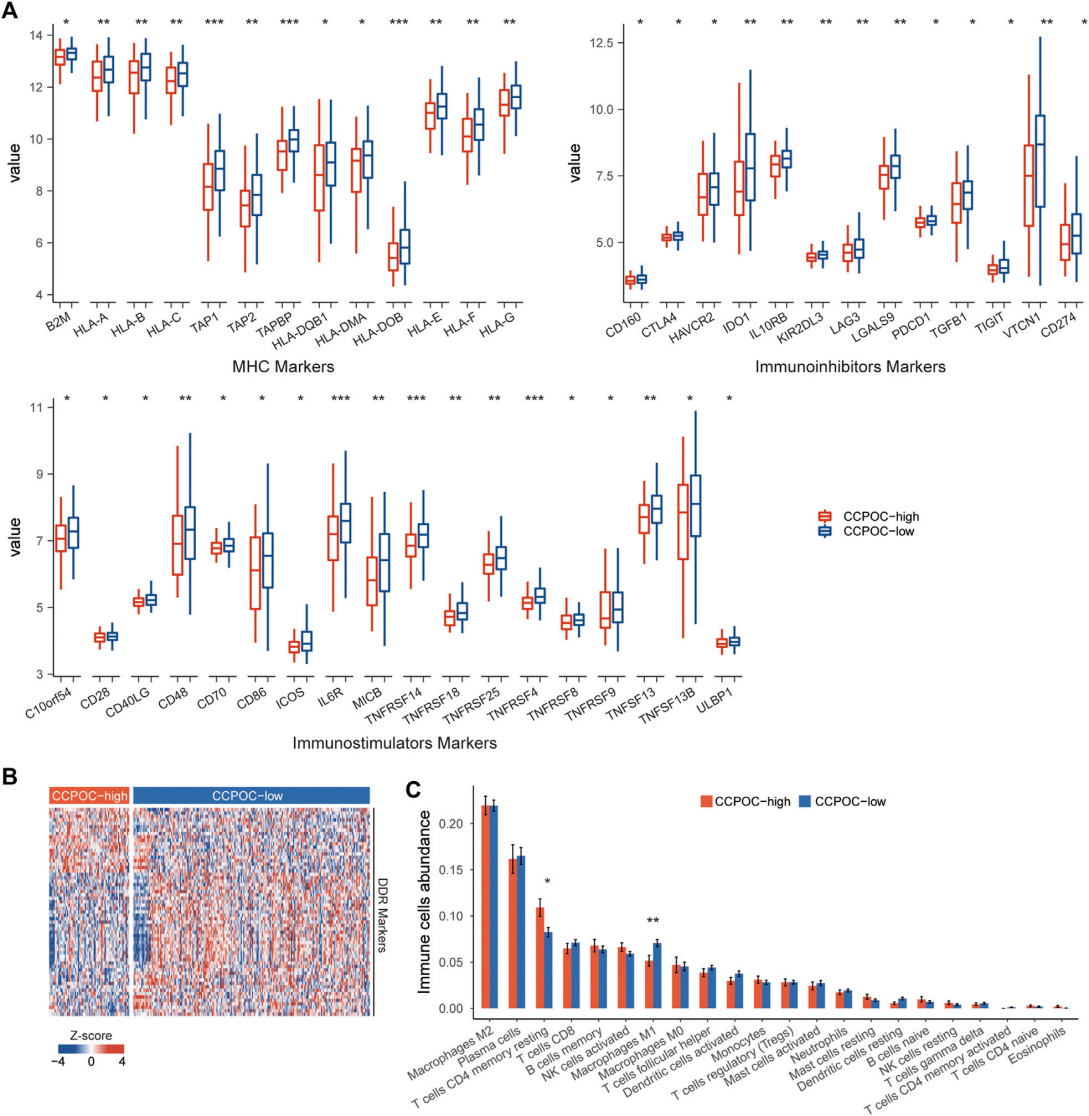
Immune microenvironment composition in CCPOC groups. **(A)** Expression levels of immuno-inhibitors, MHC I/II molecular, and immuno-stimulator markers within the CCPOC groups. **(B)** Heatmap of the expression of DDR markers between the CCPOC-high and CCPOC-low groups. **(C)** Distribution of 22 immune cells in the CCPOC groups. (*p < 0.05 and **p < 0.01).

**FIGURE 6:**
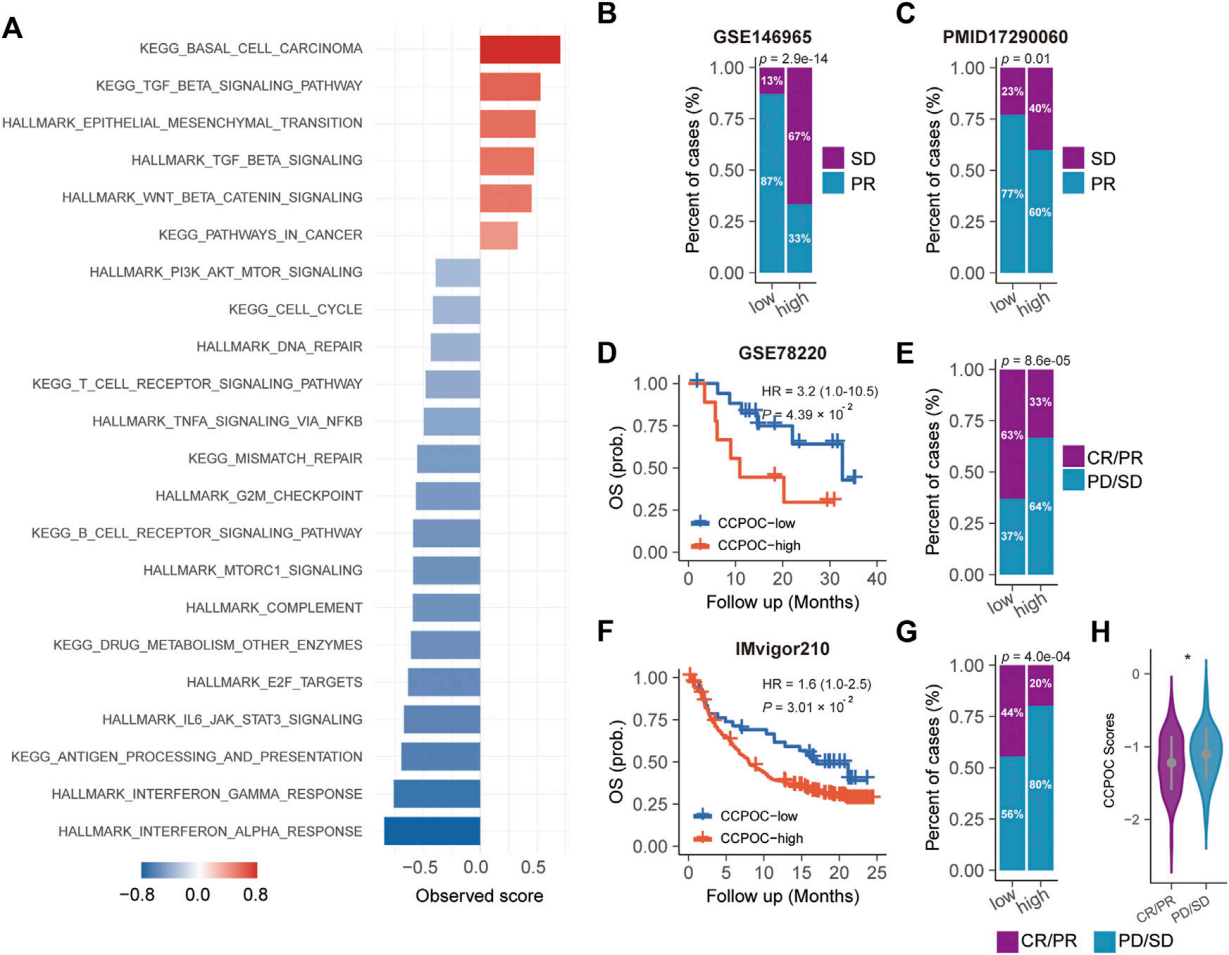
Dysregulated pathways and drug sensitivity within the CCPOC groups. **(A)** Enrichment of dysregulated pathways between the high- and low- CCPOC groups. **(B)** and **(C)** chemotherapy sensitivity analysis. Survival analyses for the CCPOC groups in the GSE78220 cohort **(D)** and the IMvigor210 cohort **(F)**. Proportion of patients with response to anti-PD-1/L1 immunotherapy in low- or high-CCPOC groups in the GSE78220 cohort **(E)** and the IMvigor210 cohort **(G)**. **(H)** Distribution of the CCPOC scores in CCPOC groups. CR, complete response; PR, partial response; SD, stable disease; PD, progressive disease.

Earlier, we showed that the CCPOC could help risk stratification of OC patients. Next, we explored the immune microenvironment composition in CCPOC-low and CCPOC-high groups. Immunomodulators have been classified into three types of molecules which include immune-inhibitors, immuno-stimulators, and major histocompatibility complex (MHC) molecules. The DNA damage response (DDR) refers to the process by which the cell maintains integrity of the genome after insult. The CCPOC-high group presented lower expression of MHC I/II molecular, immuno-inhibitor markers, immuno-stimulator markers (Figure 5A), and DDR markers (Figure 5B). The immune cell infiltration results showed that the CCPOC-high group was enriched with T cell CD4 memory resting cells (Figure 5C). Then, we conducted GSEA between the CCPOC-high and CCPOC-low groups (Supplementary Table S6). The EMT, TGF-β, and Wnt pathways were upregulated in the CCPOC-high group (Figure 6A). When analyzing CCPOC to predict chemotherapy sensitivity in the GSE146965 and PMID17290060 cohorts, chemotherapy effectiveness was lower in the CCPOC-high group than in the CCPOC-low group (Figure 6B). Immunotherapy, represented by a PD-L1/L1 blockade, has become a new breakthrough in cancer treatment. We analyzed the association between CCPOC and the response to immune checkpoint blockade therapy in two immunotherapy cohorts. In both the anti-PD-L1 cohort (IMvigor210) and the anti-PD-1 cohort (GSE78220), patients within the CCPOC-low group showed prolonged survival (Figures 6D,F). Treatment results showed that patients within the CCPOC-low group showed more efficacy against anti-PD-1/L1 immunotherapy than CCPOC-high group patients (Figure 6E and 6G-H). The aforementioned data indicate that the CCPOC-high group had lower tumor immunogenicity and lower efficacy of chemotherapy and anti-PD-1/L1 immunotherapy treatment response.

### Exploring the prognostic significance and the immune therapy response in pan-cancer

**FIGURE 7:**
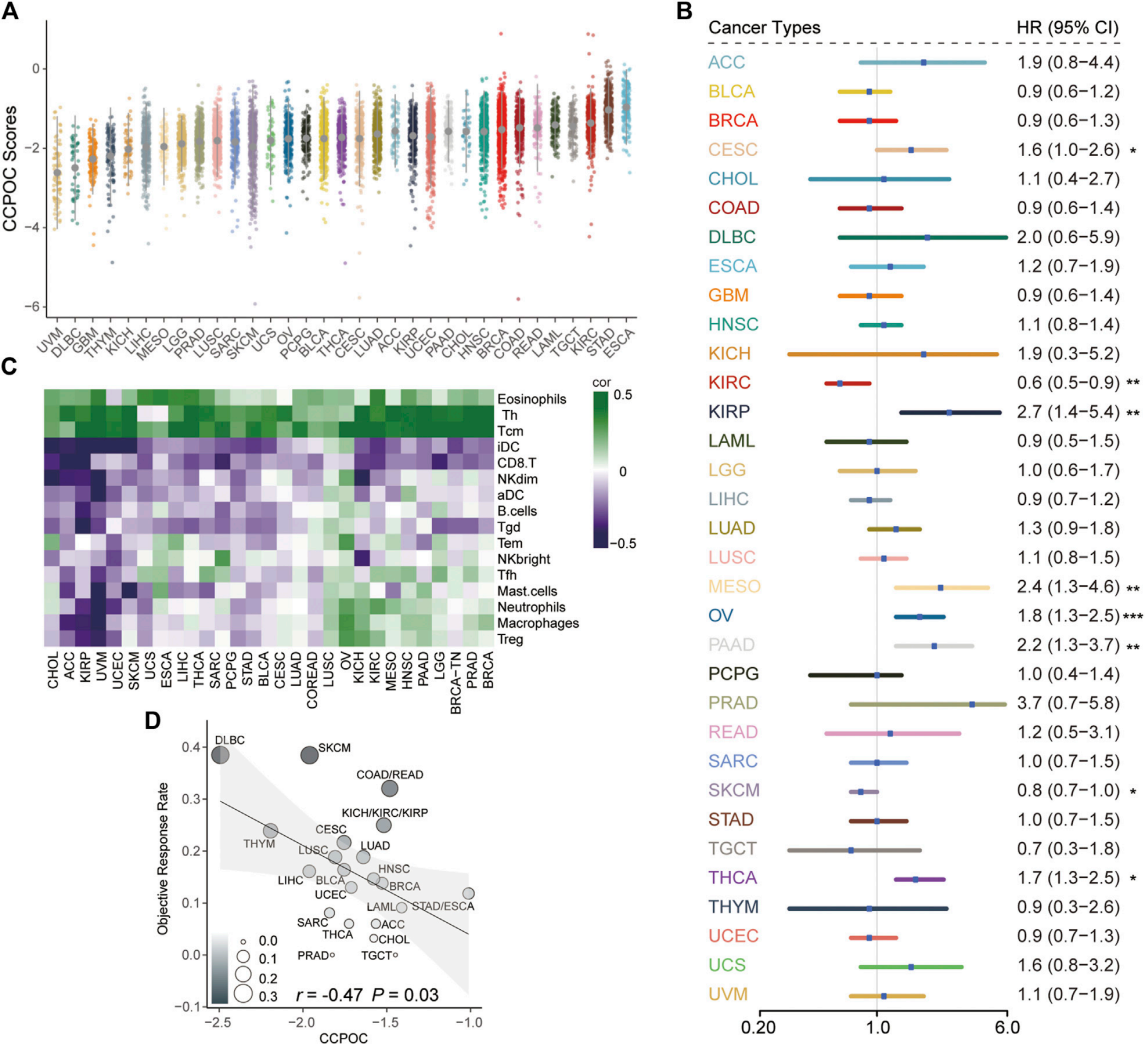
Pan-cancer analysis of the prognostic significance and anti-PD-1/PD-L1 response for CCPOC. **(A)** Distribution of CCPOC scores in pan-cancer. **(B)** Prognostic relevance of CCPOC in various cancer types. **(C)** Heatmap of the correlation of immune cells and CCPOC scores across various cancer types. **(D)** Correlation between CCPOC and anti-PD-1/PD-L1 ORR of various cancer types. (*p < 0.05, **p < 0.01, and ***p < 0.001).

Next, we explored the CCPOC signature in pan-cancer. As shown in Figure 7A, the CCPOC scores of OC were medium in the 33 cancers. Then, we explored the prognostic significance of CCPOC in pan-cancer. As presented in Figure 7B, CCPOC acted as a prognostic factor in almost 25% of cancer types. In addition, the CCPOC-high group presented low lymphocyte infiltration, such as CD8+ T cell, B cells, and NK cells (Figure 7C). Through an extensive literature search, we identified 15 tumor types for which data regarding the ORR of anti-PD-1/PD-L1 were available (OC excluded, Supplementary Table S7). In line with our suggestion, the CCPOC was negative to anti-PD-1/PD-L1 response in pan-cancer (*r* = −0.47, OC excluded) (Figure 7D). These data showed that CCPOC could be a biomarker in predicting the prognosis and anti-PD-1/PD-L1 response in other cancer types.

## Discussion

Ovarian cancer (OC) is the most lethal gynecological cancer with pathological and molecular heterogeneity characteristics (Siegel et al., 2019). Various multi-gene prognostic biomarkers have been developed (Pan and Ma, 2020; Yang et al., 2021), but their prediction efficiencies are still uncertain. Therefore, a new signature that can accurately recognize OC patients with poor prognoses is urgently needed. Based on transcriptome data, OC has been unsupervised and classified into four molecular subtypes (immunoreactive, differentiated, proliferative, and mesenchymal) with distinct molecular and clinical characteristics (Cancer Genome Atlas Research Network, 2011). The mesenchymal subtype highly expressed the mesenchymal signature and was associated with worse clinical outcomes. The prognostic signature screened based on molecular portraits specific to the worst prognosis subtype may be used for risk stratification of OC patients. In addition, many studies have explored the role of cell cycle in the prognosis prediction of tumors (Hui et al., 2021; Jiang et al., 2021). However, most studies have only studied the prognostic relevance of cell cycle without considering tumor heterogeneity. In this study, we applied a network-based approach to integrate cell cycle signature and modalities underlying the mesenchymal subtype to establish a prognostic signature termed “Cell Cycle Prognostic Signature of Ovarian Cancer” (CCPOC). To our knowledge, no prognostic cell cycle-based signature has been constructed by incorporating molecular subtyping information of OC.

The CCPOC was constructed by two cell cycle genes (POLA2 and KIF20B) which were key regulators of the mesenchymal subtype and could stratify patients into different risk groups. Within these two cell cycle genes, KIF20B can promote cell proliferation and could be a potential therapeutic target in pancreatic cancer (Chen et al., 2021). Koh et al. reported that the knockdown of POLA2 increases chemo-resistance in human lung cancer cells (Koh et al., 2016). The defined CCPOC-high group showed a worse OS than the CCPOC-low group. To confirm this finding, we validated the results in seven independent cohorts measured by various platforms and one independent internal cohort (BL-OC cohort) and found that the signature successfully stratified the prognosis in all cohorts. The CCPOC remained an independent prognostic predictor in the multivariate Cox proportional hazards analysis after adjusting for other clinical factors. In line with the findings, we found that the C-index and D-index of the CCPOC were significantly higher than those of the published prognostic models, which was superior to the current genomic classification. These data suggest that the CCPOC has a strong and reproducible prognostic value for risk stratification of OC. In addition, we also found that the CCPOC was related to weakened tumor immunogenicity and inflamed antitumor immunity, and the correlation analysis showed that CCPOC was negatively related to the ORR in pan-cancer (OC excluded), which indicated that the CCPOC-low group may be sensitive to anti-PD-1/PD-L1 therapy. Together, these findings show that the CCPOC could serve as a robust prognostic signature in OC.

This study still has some limitations. First, the prognostic signature was screened from gene expression profiles generated from microarray platforms, which are expensive, difficult to operate, and involve professional bioinformatics expertise, so it is difficult to be popularized in daily clinical application. Second, the training and validation datasets were all from retrospective studies in the study, including fresh frozen samples. Therefore, in practice, we need to detect the expression of signature genes using conventional clinical techniques, such as RT-PRC or IHC, and then reconstruct the new model and perform large-scale multicenter cohorts to validate the validity and robustness of the model.

In conclusion, using multi-dimensional network inference underlying the mesenchymal subtype of OC, we have identified and validated a two cell cycle signature, named CCPOC, to risk-stratify patients and provide an easy method for the exploration of new effective therapeutic options, including novel target drugs and immune therapy in the future.

## Data Availability

The datasets presented in this study can be found in online repositories. The names of the repository/repositories and accession number(s) can be found in the article/Supplementary Material.

## Supplementary material

SUPPLEMENTARY FIGURE S1 Kaplan–Meier analysis of different molecular subtypes.

SUPPLEMENTARY FIGURE S2 Expression levels of POLA2 and KIF20B **(B)** genes among different subtypes.

SUPPLEMENTARY FIGURE S3 2-year and 5-year AUC of the CCPOC score in the BL-OC cohort.

SUPPLEMENTARY FIGURE S4 Heatmap of the expression of the two candidate immune genes.

SUPPLEMENTARY FIGURE S5 Kaplan–Meier analysis of CCPOC risk groups within different conditions.
