# Supplementary figures and images for: Integrating cell cycle score for precise risk stratification in ovarian cancer
Source: Front Genet. 2022 Aug 17;13:958092. doi: 10.3389/fgene.2022.958092 (PMC9428269; doi:10.3389/fgene.2022.958092)

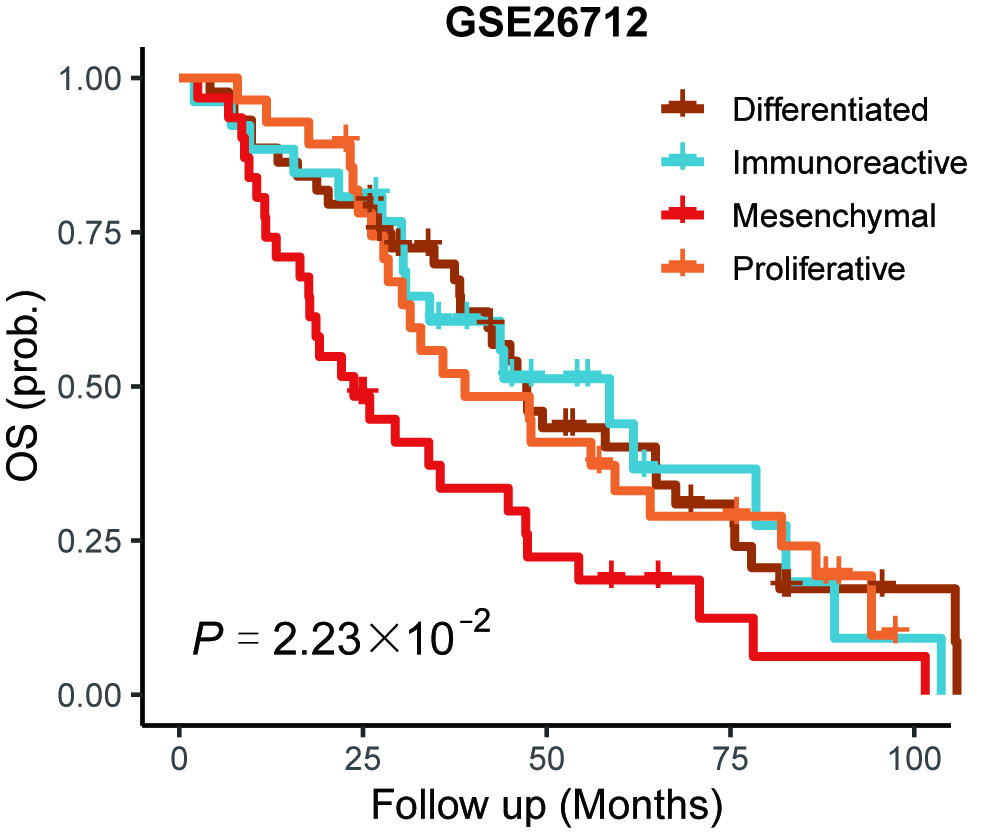

Supplement: Supplementary file 1 [file DataSheet1.ZIP › Supplementary Figure S1.tif]

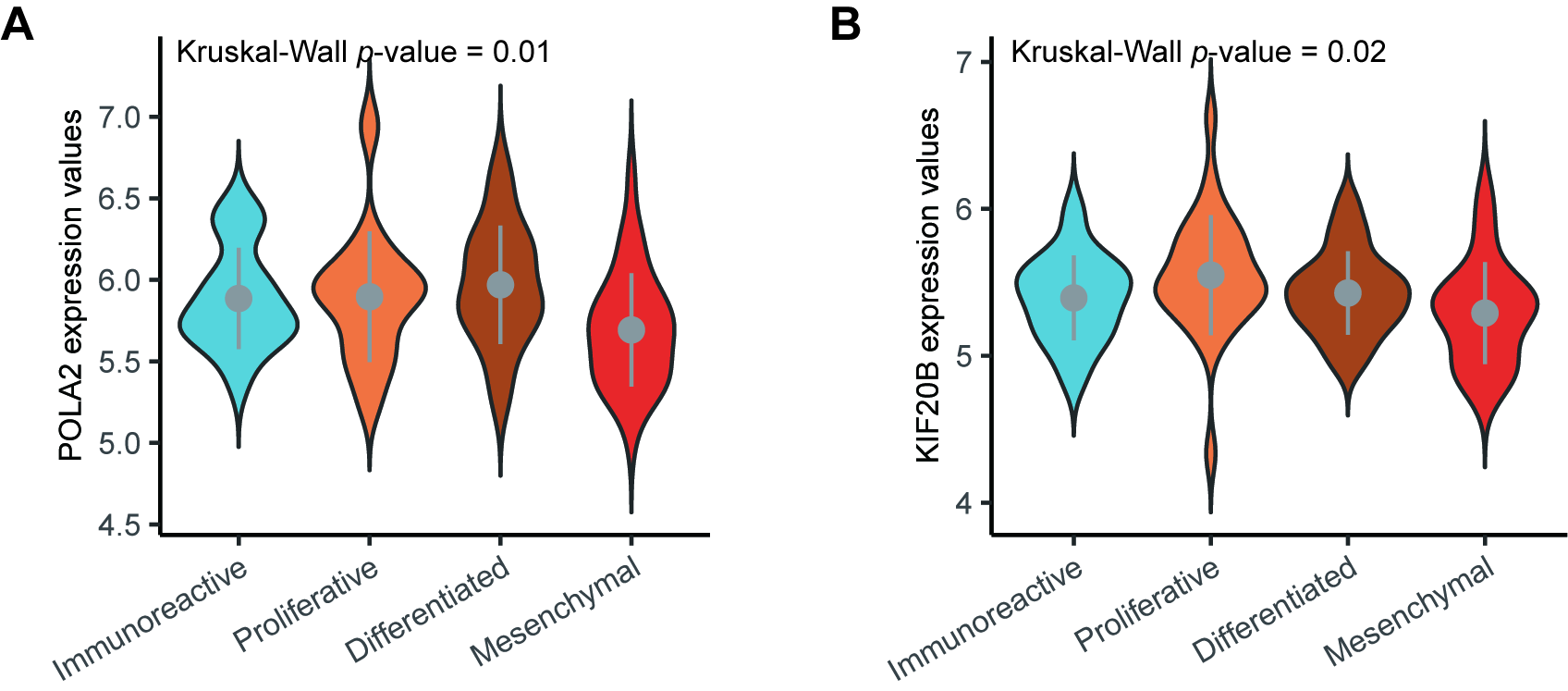

Supplement: Supplementary file 1 [file DataSheet1.ZIP › Supplementary Figure S2.tif]

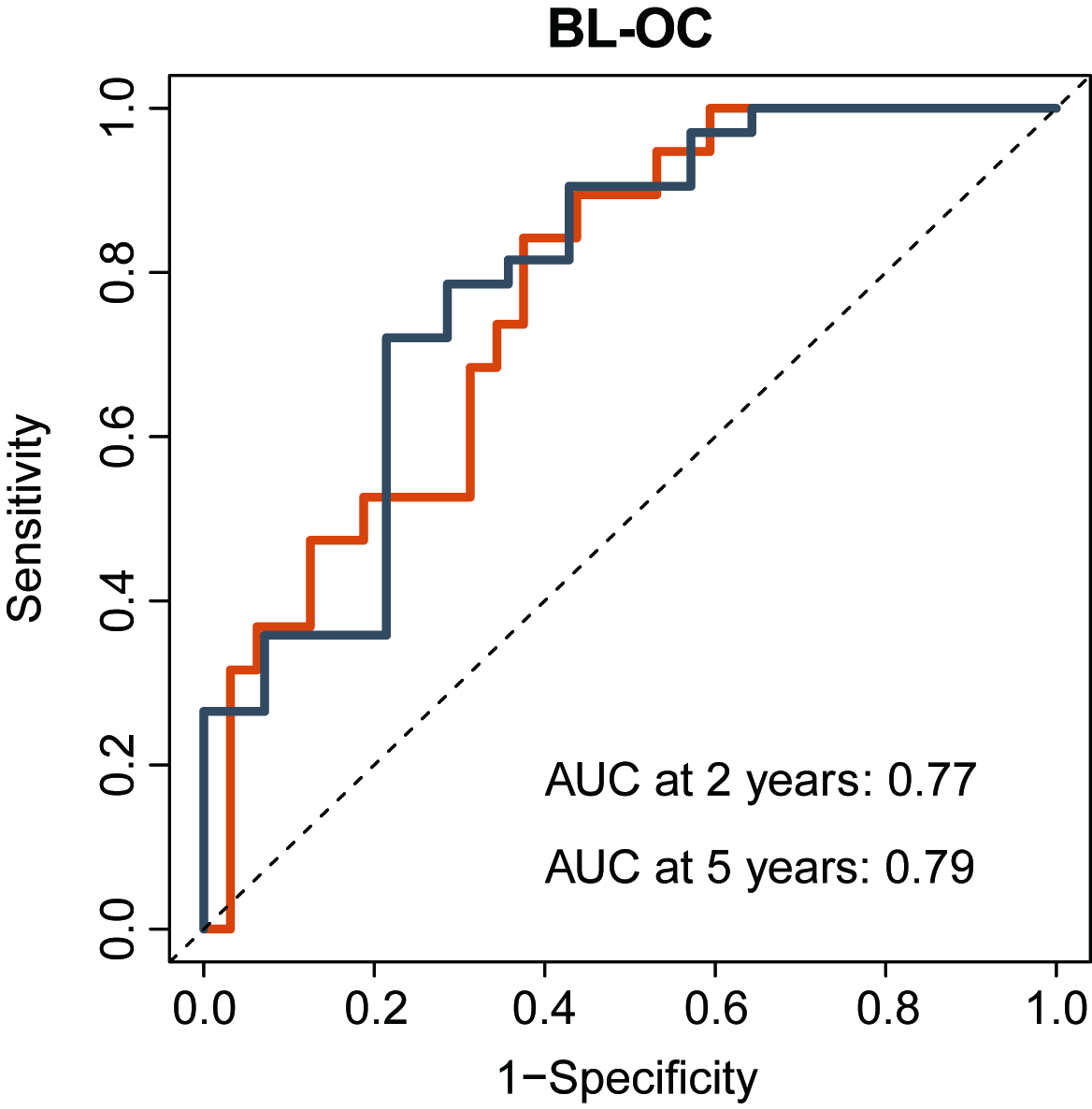

Supplement: Supplementary file 1 [file DataSheet1.ZIP › Supplementary Figure S3.tif]

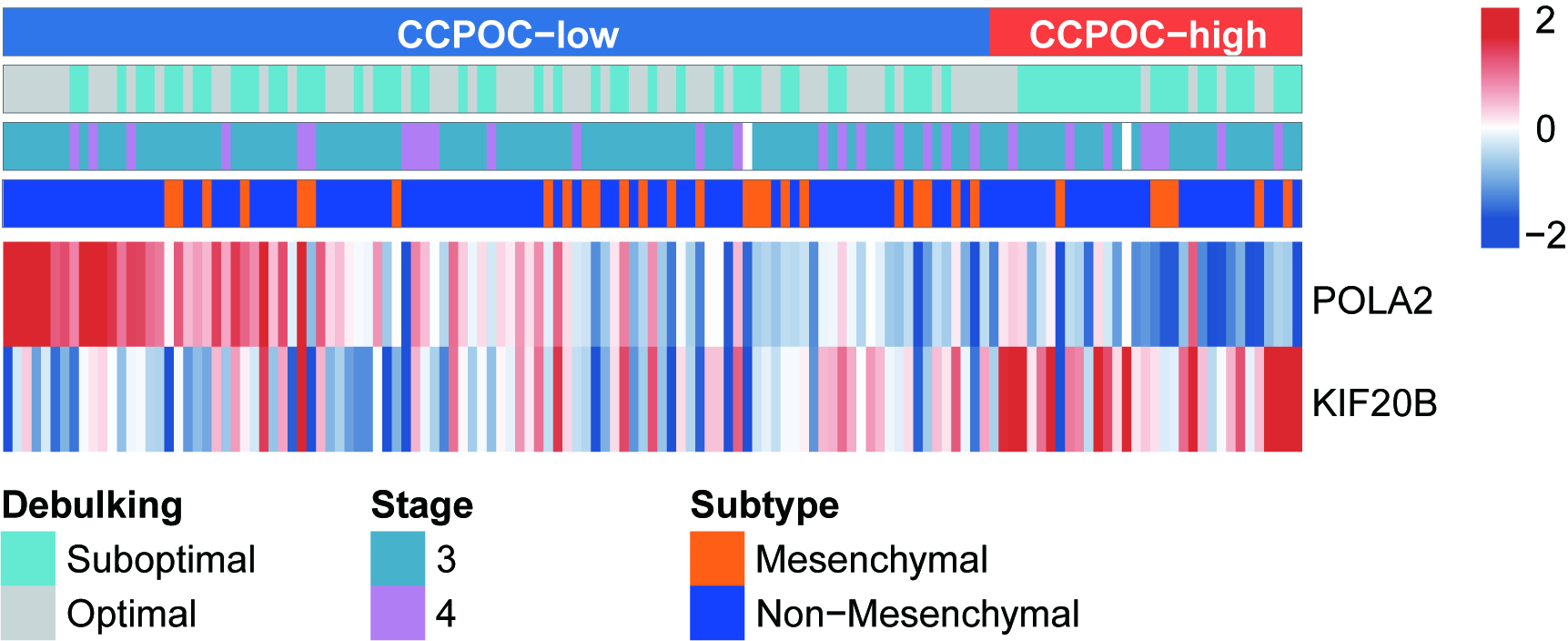

Supplement: Supplementary file 1 [file DataSheet1.ZIP › Supplementary Figure S4.tif]

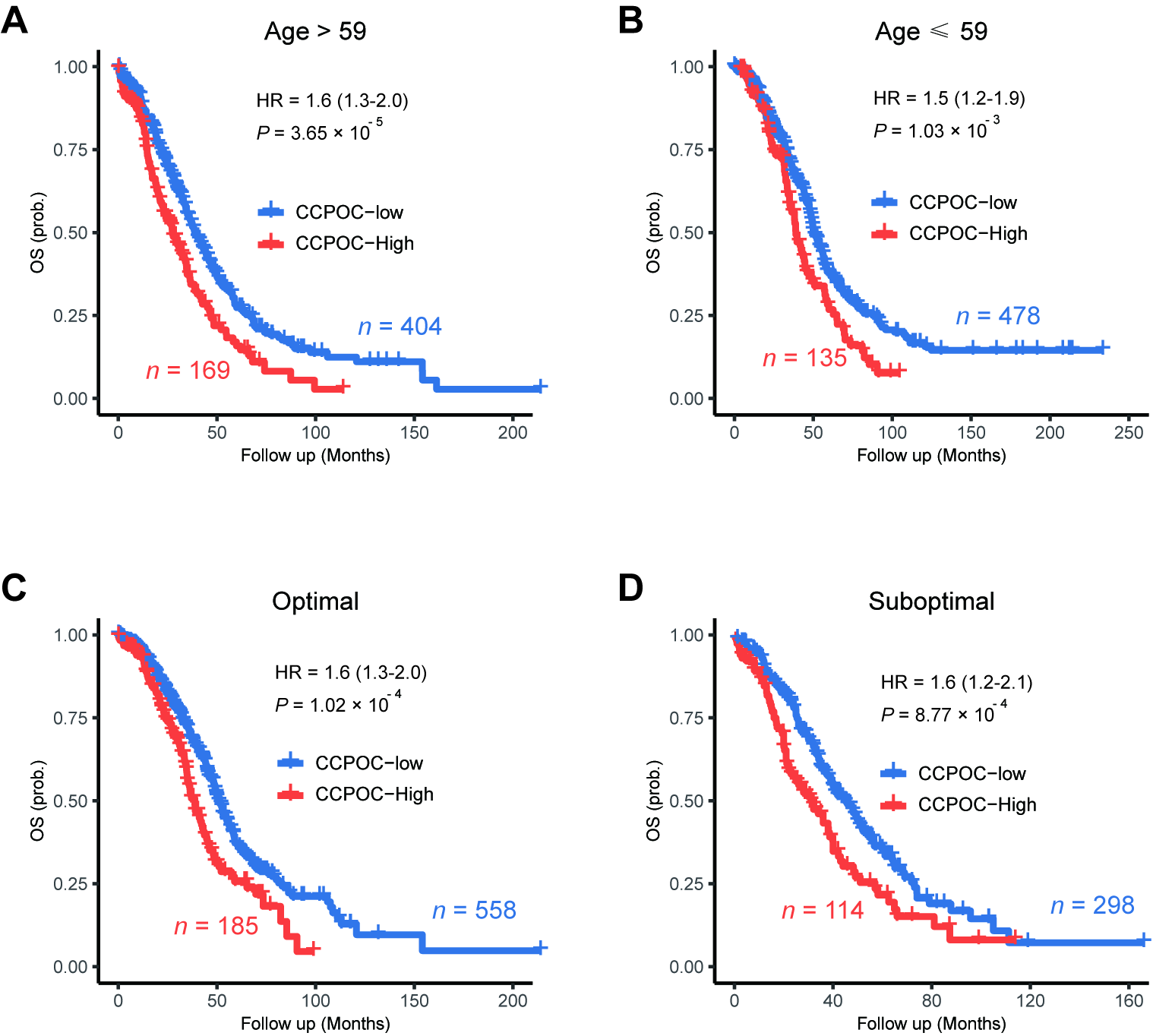

Supplement: Supplementary file 1 [file DataSheet1.ZIP › Supplementary Figure S5.tif]
